# Postpartum septic pelvic thrombophlebitis and ovarian vein thrombosis after caesarean section: a rare case report

**DOI:** 10.1186/s12884-021-04037-4

**Published:** 2021-08-17

**Authors:** Qin Shi, Deborah Shulamite Gandi, Yurong Hua, Yi Zhu, Jinhan Yao, Xiaoqing Yang, Yunzhao Xu, Yuquan Zhang

**Affiliations:** 1 Department of Obstetrics and Gynecology, Affiliated Hospital of Nantong University, Nantong, People’s Republic of China; 2 Affiliated Maternity and Child Health Care Hospital of Nantong University, Nantong, China

**Keywords:** Septic pelvic thrombophlebitis, Ovarian vein thrombosis, Caesarean section, Case report

## Abstract

**Background:**

Septic pelvic thrombophlebitis (SPT) is a well-recognized but rare puerperal complication that has two types: ovarian vein thrombophlebitis (OVT) and deep septic pelvic thrombophlebitis (DSPT). The present case report describes the clinical and imaging findings of a female patient diagnosed with right ovarian vein infectious thrombophlebitis after caesarean section (C-section).

**Case presentation:**

A 35-year-old G3P2 female who presented with a foetal vein Galen malformation at 41 weeks of gestation underwent C-section. The patient had high fever after C-section, and anti-inflammatory treatment was not effective within 1 week. An abdominal wall incision haematoma was found, and a second surgery for the removal of the abdominal wall haematoma was performed. The patient was ultimately diagnosed with abdominal incision haematoma and right ovarian vein infectious thrombophlebitis after C-section. We used imipenem and tigecycline to strengthen the anti-inflammatory effects, simultaneously administrating low-molecular-weight heparin and warfarin as anticoagulant therapy. On the 30th day after C-section, the right ovarian vein thrombus disappeared.

**Conclusion:**

This case illustrates the need to consider the potential relationship between abdominal incision haematoma and ovarian vein thrombophlebitis. Despite advances in the management of venous thromboembolism globally, more data on epidemiology in terms of first incidence, prevalence, recurrence and risk factors, management of bleeding complications, and increased awareness in Asian populations are necessary.

## Background

Septic pelvic thrombophlebitis (SPT) is a well-recognized but rare puerperal complication (approximately 1 in 3000 deliveries). It occurs more frequently after caesarean section (C-section) (1 in 800 cases) than after vaginal delivery (1 in 9000 cases), probably due to the higher rate of puerperal infection [1, 2]. This complication was quite prevalent previously; its management was almost solely based on surgical treatment, and it was associated with high mortality rates. Recently, the prognosis of SPT has improved, but it can still cause life-threatening conditions [3]. There are two types of SPT: ovarian vein thrombophlebitis (OVT) and deep septic pelvic thrombophlebitis (DSPT). Although these types may differ in clinical presentation and diagnostic findings, they have common pathogenic mechanisms and often occur together. SPT is an important differential diagnosis of abdominal pain and fever in the postpartum period, and its diagnosis might be challenging. The present case report describes the clinical and imaging findings of a female patient diagnosed with right ovarian vein infectious thrombophlebitis after C-section.

## Case presentation

A 35-year-old G3P2 female presented with a LOA (left occipital anterior) foetal position at 41 weeks of gestation. In 2001, she underwent an appendectomy. In 2001, she underwent an appendectomy. In 2008, she delivered a male newborn with a birthweight of 3500 g. In 2010, she underwent a medical abortion during early pregnancy. Prior to this pregnancy, both gynaecological examination and transvaginal ultrasonography were negative. Her menstrual cycle was regular. Her pregnancy at this time was complicated by a large foetus size and abnormal foetal movements, as detected by ultrasound examination, which suggested a Vein of Galen malformation. She was admitted with a blood pressure of 106/70 mmHg, and she denied headache, abdominal pain, or oedema. Reflexes were normal. Her height was 168 cm, her weight was 72 kg, and her body mass index (BMI) was 25.5 kg/m^2^. Her haemoglobin level was 130 g/L. The patient was delivered by caesarean section on the second day with intraoperative blood loss of 350 ml. We delivered a male newborn with a birthweight of 4600 g and an APGAR score of 10/10/10.

On postoperative day 1, the patient complained of the surgical wound being painful but bearable. Physical examination showed normal body temperature with dry and odourless petechial haemorrhage on the skin surrounding the abdominal incision above the uterine margin and below the navel. The white blood cell (WBC) count was 10.20 × 10^9^/L, the haemoglobin level was 97 g/L, and the platelet count level was 79 × 10^9^/L. On postoperative day 2, she complained of aggravated pain at the abdominal incision and odourless ecchymosis of the skin around the abdominal incision at the right lateral margin with local tension. The body temperature was 37.5 °C, the WBC count was 11.32× 10^9^/L, the haemoglobin level was 88 g/L, and the platelet count level was 85 × 10^9^/L. A palpable mass with unclear boundary and tenderness was detected. On postoperative day 3, the pain of the patient’s incision progressively aggravated, with a body temperature of 38.2 °C. An ultrasound examination revealed an abdominal wall incision haematoma with a size of approximately 43 × 32 × 10 mm. The WBC count was 9.05× 10^9^/L. The haemoglobin level was 83 g/L, and the platelet count was 88 × 10^9^/L.

**Fig. 1:**
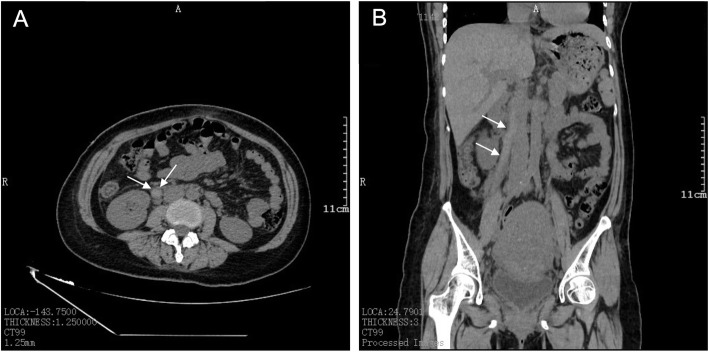
CT image showing right ovarian vein thrombosis. **A** Superior-inferior view. **B** Anterior-posterior view

**Fig. 2:**
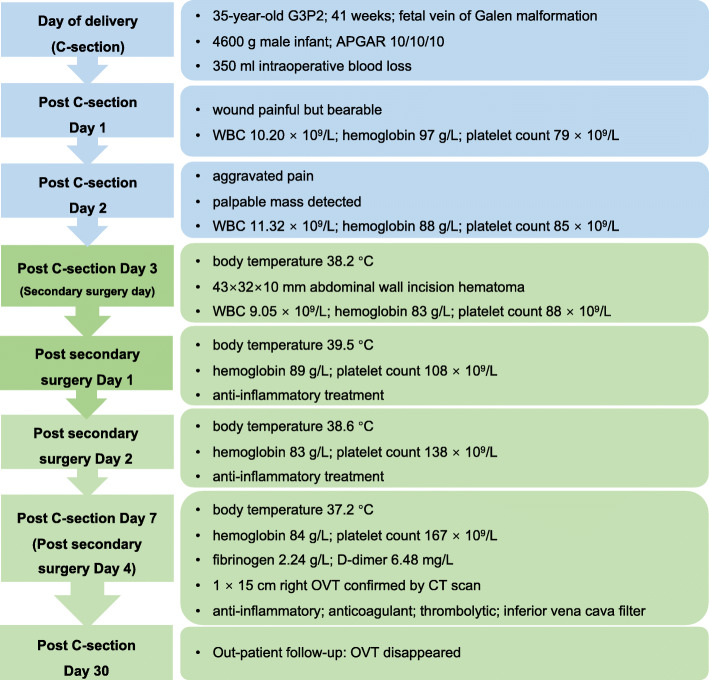
Timeline of events and findings. WBC: white blood cell; OVT: ovarian vein thrombophlebitis

After 1–3 days of C-section, the patient’s haemoglobin level showed a progressive decline from 97 to 88 to 83 g/L. The platelet count had increased from 79 to 88 × 10^9^/L. B-ultrasound imaging suggested abdominal wall incision haematoma. Based on the abovementioned findings, we considered the diagnosis of abdominal incision haematoma after C-section. A second surgery for the removal of the abdominal wall haematoma was performed on the third day after C-section. We removed a blood clot of approximately 100 ml between the rectus abdominis and the anterior sheath. Moreover, approximately 10 ml of blood clot was removed from the right rear of the rectus abdominis, peritoneal front. The intraoperative blood loss was approximately 20 ml. On postoperative day 1 of the second surgery, the patient recorded a body temperature of 39.5 °C, low haemoglobin level of 89 g/L, and increased platelet count of 108 × 10^9^/L. The patient was administered piperacillin tazobactam at 2.5 g q 8 h and levofloxacin at 0.4 g q d. Acetaminophen was also administered as an antipyretic symptomatic treatment. The patient was also informed to get out of bed as early as possible and move more frequently to prevent thrombosis. On postoperative day 2 of the second surgery, the body temperature was 38.6 °C, the haemoglobin level was 83 g/L and the platelet count increased to 138 × 10^9^/L. On postoperative day 3, the body temperature declined to 37.2 °C. On postoperative day 4 of the second surgery, the haemoglobin level was 84 g/L, and the platelet count level was 167 × 10^9^/L. Her fibrinogen level was 2.24 g/L, and her D-dimer level was 6.48 mg/L, which suggested thrombosis. A computed tomography (CT) scan confirmed the diagnosis of right OVT, with a size of approximately 1 × 15 cm (Fig. 1). The patient was then placed on imipenem and tigecycline therapy to strengthen the anti-inflammatory response, with simultaneous administration of low-molecular-weight heparin and warfarin as an anticoagulant therapy. The timeline from diagnosis to treatment is shown in Fig. 2.

On the 10th day after C-section, the patient was transferred to a superior hospital for inferior vena cava filter implantation and subsequent anticoagulant and thrombolytic therapy. On the 30th day after C-section, a CT scan revealed that the right OVT had disappeared.

## Discussion and conclusions

In this case, we reported that right ovarian vein infectious thrombophlebitis occurred in a patient with foetal Vein of Galen malformation and abdominal incision haematoma after C-section. Because of the low incidence of SPT, it is easily ignored by clinicians. This patient had a high fever and abnormal blood examination findings after C-section. Anti-inflammatory treatment was not effective after 1 week. She underwent a second surgery for the removal of the abdominal wall incision haematoma to control the condition. It remains to be determined whether the haematoma was related to right ovarian infectious thromboembolic phlebitis. Wang et al. [4] concluded that venous thromboembolism (VTE) has been considerably underestimated in Asia. Limited data exist on the incidence of VTE in the current literature.

An important feature of SPT is the simultaneous presence of bacterial infections and venous thrombosis [5]. The affected vein is characterized by phlebitis. The patient has severe fever or high fever, and a white blood cell rise is common in puerperal infections in pelvic infectious thrombophlebitis. SPT has dangerous clinical manifestations. In addition to pelvic infections, there can also be severe symptoms of high fever and poisoning. If the infected thrombus breaks off, it can lead to lung abscesses and abscesses in other parts of the body.

Slow blood flow, damaged inner wall of the vein, and blood hypercoagulation are three major factors that increase thrombotic inflammation. When the infection is combined, the bacterial decomposition of heparinase decomposes heparin to promote coagulation. Pelvic venous thrombosis and bacterial infection are two essential factors for SPT [6]. However, the sequence and causal relationship between the two are still unknown. In a damaged venous wall, in the hypercoagulate state of postpartum blood, an infectious thrombosis is formed [7].

The dimer is produced by pyrolysis of plasminogen by plasmin. It can reflect the degree of fibrinolysis of the early activation of the coagulation reaction, and it is a specific marker of activation of the coagulation and fibrinolysis system [8]. Regular inspection of D-dimer content may be an important method for early elimination of thrombotic diseases [9]. D-dimer levels > 500 μg/L are the diagnostic criteria, with a sensitivity of 96.8% and a specificity of only 35.2% [10]. Therefore, this method cannot be used to diagnose thrombosis except for in certain diseases.

In view of the lack of precise laboratory examination methods, the diagnosis of SPT has been challenged. After physical examination, the patient usually has no toxic reaction. The lower abdomen may have tenderness on palpation, and occasionally, a cord or sausage-like abdominal tenderness may be recognized [11]. This is the most diagnostic finding in an abdominal examination, but it is rare. In less than one-third of cases, leukocytosis is moderate, and blood cultures are positive. In this case, although the patient’s clinical condition did not change, the analysis parameters of inflammation improved after the third antibiotic treatment.

Although the incidence of VTE in Asian populations is lower than that in Western countries, the overall burden of VTE in Asia has been greatly underestimated [4]. Factors that can explain the lower prevalence of VTE in Asian populations relative to Western populations may include the limited availability of Asian epidemiological data, ethnic differences in genetic susceptibility to VTE, low awareness of thrombotic diseases in diagnosis, and fewer Asian patients with symptomatic VTE [12]. Generally, the clinical evaluation, diagnostic tests and treatment precautions of VTE between Asian and Western populations are the same. Management of VTE is based on balancing the benefits of treatment with the risk of bleeding. For Asian populations, this is a particularly important consideration because of the increased risk of intracranial haemorrhage caused by the use of vitamin K antagonists [13]. In major phase 3 clinical trials that include Asian populations, non-vitamin K antagonist oral anticoagulants (NOACs) have shown advantages over current treatment modalities in terms of bleeding outcomes [14]. Although the management of VTE has progressed, the management data of the incidence, prevalence, recurrence rate, risk factors and bleeding complications in the Asian population are still limited, and awareness needs to be improved. The decision to manage VTE is based on a balance between the benefits of treatment and the risk of bleeding. This is a particularly important consideration for the Asian population because Asians tend to have increased bleeding, especially intracranial haemorrhage [15]. Considering this risk, it is important to diagnose the disease in a timely and accurate manner and safely exclude the disease when it does not exist.

The case presented here illustrates the need to consider the potential relationship between abdominal incision haematoma and ovarian vein thrombophlebitis. Although progress has been made in the management of venous thromboembolism on a global scale, more epidemiological data are still needed, including first incidence, prevalence, recurrence and risk factors and management of bleeding complications in Asian populations.

## Data Availability

All data are available in the manuscript.

## Abbreviations

SPT: Septic pelvic thrombophlebitis
PVT: Pelvic venous thrombosis
VTE: Venous thromboembolism
PT: Pelvic thrombophlebitis
OVT: Ovarian vein thrombosis
DSPT: Deep septic pelvic thrombophlebitis
NOACs: Non-vitamin K antagonist oral anticoagulants
